# Photomodulated Extrusion as a Localized Endovascular Hydrogel Deposition Method

**DOI:** 10.1002/adhm.202202632

**Published:** 2023-02-03

**Authors:** Yuta Dobashi, Jerry C. Ku, Joel Ramjist, Christopher Pasarikovski, Konrad Walus, John D. W. Madden, Victor X. D. Yang

**Affiliations:** ^1^ Institute of Medical Science University of Toronto Toronto Ontario M5S 1A1 Canada; ^2^ Sunnybrook Research Institute Toronto Ontario M4N 3M5 Canada; ^3^ Division of Neurosurgery Department of Surgery University of Toronto Toronto Ontario M5S 1A1 Canada; ^4^ Department of Electrical and Computer Engineering School of Biomedical Engineering University of British Columbia Vancouver British Columbia V6T 1Z4 Canada

**Keywords:** drug delivery, endovascular embolization, hydrogels, shear thinning

## Abstract

Minimally invasive endovascular embolization is used to treat a wide range of diseases in neurology, oncology, and trauma where the vascular morphologies and corresponding hemodynamics vary greatly. Current techniques based on metallic coils, flow diverters, liquid embolics, and suspended microspheres are limited in their ability to address a wide variety of vasculature and can be plagued by complications including distal migration, compaction, and inappropriate vascular remodeling. Further, these endovascular devices currently offer limited therapeutic functions beyond flow control such as drug delivery. Herein, a novel in situ microcatheter‐based photomodulated extrusion approach capable of dynamically tuning the physical and morphological properties of injectable hydrogels, optimizing for local hemodynamic environment and vascular morphology, is proposed and demonstrated. A shear thinning and photoactivated poly(ethylene glycol diacrylate)‐nanosilicate (PEGDA‐nSi) hydrogel is used to demonstrate multiple extrusion modes which are controlled by photokinetics and device configurations. Real‐time photomodulation of injected hydrogel viscosity and modulus is successfully used for embolization in various vasculatures, including high‐flow large vessels and arterial‐to‐arterial capillary shunts. Furthermore, a generalizable therapeutic delivery platform is proposed by demonstrating a core–shell structured extrusion encapsulating doxorubicin to achieve a more sustained release compared to unencapsulated payload.

## Graphical abstract

A novel microcatheter‐based photomodulated extrusion device is proposed. An optical waveguide integrated microcatheter is used to localize shear‐thinning and photosensitive hydrogel in a variety of endovascular anatomies. Using a dual lumen configuration, extrusion of core–shell hydrogels encapsulating doxorubicin is demonstrated. A generalizable therapeutic platform is proposed, including drug delivery, tissue engineering, and endovascular embolization.

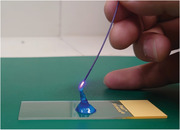

## 1 Introduction

Vascular diseases such as hypervascular tumors, hyperplastic conditions, hemorrhagic blood vessels, as well as vascular abnormalities including aneurysms and arterio‐venous malformations (AVMs) have historically been treated using traditional open surgical techniques. Over the past 20 years, however, there has been a rapid shift toward minimally invasive techniques either as a primary or adjunct treatment wherein intravascular catheters are navigated to the disease site to deploy a variety of flow control devices to re‐direct or block blood flow.^[^ ^1^, ^2^ ^]^ Despite the benefits of minimally invasive approaches such as shorter recovery times, current selection of flow control devices, such as metallic coils, flow diverting stents, gelatin foams, suspended microspheres, and liquid embolics have respective disadvantages that limit safety and efficacy. Metallic coils, used to occlude aneurysms and various vessels, only provide a low‐density occlusion and rely on body's autologous clot formation, making them susceptible to structural compaction leading to reperfusion and distal migration in ≈25% and 2.5% of the cases, respectively.^[^ ^3^, ^4^, ^5^, ^6^ ^]^ Sufficient packing density is difficult to achieve in larger aneurysms while excessive packing densities risk vessel wall rupture. Flow diverters bridging over aneurysmal necks suffer from thrombogenicity and require lifelong antiplatelet therapy.^[^ ^7^ ^]^ Further, the limited conformability of flow diverters often results in malapposition against the vessel walls leading to flow leakage and endothelial hyperplasia.^[^ ^7^ ^]^ Liquid embolics based on cyanoacrylate or precipitated hydrophobic polymers create a conformal plug within the vascular geometry and a robust seal even in moderate to high flow scenarios such as AVMs and dural fistulas. However, liquid embolic products such as Trufill and Onyx suffer from delivery challenges due to rapid and uncontrollable solidification mechanisms employed, in which the delivery catheter can be clogged and subsequently bonded to the embolic released into the vasculature due to premature solidification, or conversely result in distal non‐target embolization in the case of excessive injection rate.^[^ ^8^, ^9^, ^10^ ^]^ Moreover, arterial injection of dimethyl sulfoxide (DMSO), a commonly used solvent for precipitating polymer embolics, has been associated with vasospasms and acute angiotoxicity leading to necrosis.^[^ ^9^, ^11^, ^12^ ^]^ Polymerization of cyanoacrylate releases formaldehyde, which can result in chronic vascular inflammation.^[^ ^13^, ^14^ ^]^ Use of Onyx for aneurysm treatments theoretically improve upon traditional coils with superior packing densities thanks to material conformability. In practice, however, incomplete fills and recanalization are not uncommon.^[^ ^15^, ^16^ ^]^ Suboptimal injection and solidification of Onyx in AVM nidus or aneurysm sac can result in hemorrhage or downstream ischemic infarct.^[^ ^17^, ^18^ ^]^ Injectable suspended microspheres rely on controlled accumulation in capillary beds to embolize. While they are more easily injected compared to the precipitating polymers and are cheaper, they are generally more susceptible to recanalization in high flow cases such as in brain AVMs that extensively bypass capillaries^[^ ^19^ ^]^ and are only suitable for terminal microvascular structures. In some cases such as tumor embolization, intravascular local therapeutic deposition is a desirable adjunct capability.^[^ ^20^, ^21^ ^]^ For instance, drug eluting microspheres have been clinically available for simultaneous tumor embolization and doxorubicin delivery following resection and percutaneous ablation; however, the large variety of embolic sizes, delivery conditions, and outcomes have made it difficult to understand the efficacies of the drug elution.^[^ ^22^, ^23^, ^24^ ^]^ Broader interests are arising for local therapeutic depots to combat not only oncological diseases but also atherosclerosis, myocardial infarcts, spinal cord injuries, and more.^[^ ^25^, ^26^, ^27^, ^28^ ^]^

Injectable hydrogels have been employed in numerous biomedical applications such as scaffolds for tissue engineering^[^ ^29^, ^30^ ^]^ and localized drug delivery.^[^ ^31^ ^]^ Recently, more challenging intracatheter endovascular deployment of injectable hydrogels has been envisaged for both vascular embolization and drug delivery applications.^[^ ^26^ ^]^ Physical design of catheter‐injectable hydrogels is particularly limited by the precursor viscosity and the sol–gel transition mechanisms.^[^ ^32^, ^33^, ^34^ ^]^ Low viscosity precursors are necessary to avoid excessive pressure and shear forces during injection through microcatheters whose lengths can exceed 150 cm. Gelation must be accurately timed to occur promptly at the injection site, where the blood flow makes material localization challenging. Previously proposed hydrogel based embolics have utilized time‐dependent and irreversible gelation mechanisms,^[^ ^32^, ^33^, ^34^, ^35^, ^36^ ^]^ which can impart a constraint on the unpredictable nature of clinical workflow in a pulsatile circulatory environment. Shear thinning and self‐healing hydrogels have gained popularity for their injectability and rapid recovery of modulus at the deposition site.^[^ ^37^, ^38^, ^39^ ^]^ However, the weakly bound networks may be unsuitable for physically demanding applications such as cerebral AVMs, fistulas, and aneurysms requiring resistance to high hemodynamic pressure and shear. Reaching such distal locations would require the nominal viscosity of such material to be reduced to the point that they may not retain sufficient hemostatic ability in such scenarios. Such limitations naturally justifies recent interests in utilizing photopolymerization as the in situ crosslinking mechanism for hydrogel based embolics.^[^ ^40^, ^41^ ^]^ This approach is attractive as it can help overcome the tradeoff between precursor viscosity and the mechanical strength of the resultant gel, which is nontrivial particularly in distal blood vessels requiring microcatheters.

In this work, we hypothesize that photocrosslinking can enhance the versatility of a shear thinning hydrogel by adding a secondary solidification mechanism that is readily controllable through digital modulation of optical power. We thus design a shear‐thinning and UV‐activated hydrogel based on poly(ethylene glycol diacrylate) (PEGDA) and silicate nanoplatelet (nSi) that is both conformable and swelling resistant, suitable for surviving in hemodynamic environments long term. We further develop, characterize, and optimize a custom waveguide‐integrated microcatheter capable of photomodulated extrusion, enabling distal deposition of hydrogels with varying mechanical properties from a single precursor. We show early efficacy of the proposed technology in porcine model embolization where a superior vascular conformity and stability of the injected hydrogel was observed in a variety of anatomies. Finally, we show a dual‐lumen configuration of the catheter to extrude a core–shell hydrogel, encapsulating doxorubicin for a more sustained release than unencapsulated payload, paving way to a scalable drug delivery method.

## 2 Results

### 2.1 Design and Formulation of PEGDA‐nSi Hydrogels

**Figure 1:**
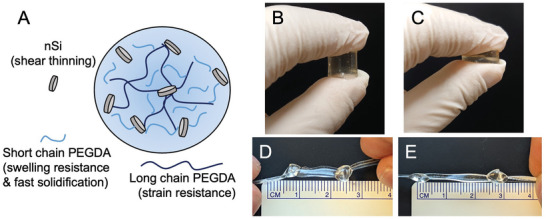
Design of the PEGDA‐nSi hydrogel. A) PEGDA‐nSi comprises of short‐chain PEGDA (*M*_w_ = 700) and long‐chain PEGDA (*M*_w_ = 10 000), which provide the hydrogel with rapid crosslinking, swelling resistance, and strain resistance properties. The nSi reversibly interact with PEGDA via electrostatic forces, making the precursor shear thinning. The resulting crosslinked hydrogel is photographically shown to withstand B,C) high compressive strain and D,E) high tensile strain, as indicated by the distances of the knots.

PEGDA based hydrogels have previously been shown to be versatile and represent the basis for bioprinting, tissue engineering, and drug delivery among other applications.^[^ ^29^, ^42^, ^43^ ^]^ nSi is a disc shaped (1 nm thick, ≈10 nm diameter) synthetic clay bearing a negative charge on its surface and is expected to reversibly interact with the water molecules and polymer chains, as well as greatly enhance the mechanical strength when crosslinked.^[^ ^44^ ^]^ nSi has been of interest for its ability to act as the carrier for various therapeutics and as the site of cellular attachment and proliferation.^[^ ^44^, ^45^ ^]^ Herein, low viscosity hydrogel precursors (herein abbreviated as PEGDA‐nSi) were formulated based on PEGDA (*M*_w_ = 700, 10k). Briefly, a total of 15% w/v of PEGDA were combined with 2–4% w/v of Laponite XLG nSi in deionized water. Short (700) and long (10k) chains were blended in order to attain a combination of swelling resistance, rapid photocrosslinking, and conformability/strain resistance (**Figure** **1**A). The crosslinked PEGDA‐nSi hydrogel is shown to be highly mechanically robust, withstanding large (≈50%) compressive strains without fracture (Figure 1B,C) among other manipulations by hand, such as being tied into a knot and stretched by 100% when the hydrogel is formed into a string (Figure 1D,E).

### 2.2 Rheological and Mechanical Properties of PEGDA‐nSi Hydrogels

**Figure 2:**
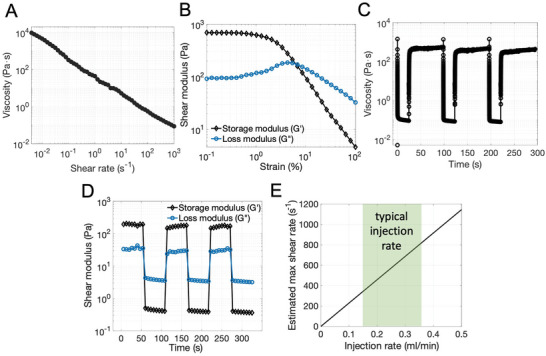
Characterization of PEGDA‐nSi hydrogel, showing rheological suitability for microcatheter injection and mechanical suitability for withstanding hemodynamic forces. A) Shear rate sweep of PEGDA‐nSi precursor, showing the precursor's shear thinning property. B) Strain sweep of PEGDA‐nSi precursor, showing linear viscoelasticity over a moderate range of up to several percent strain. C) Step‐shear measurement of PEGDA‐nSi precursor. D) Step‐strain measurement of PEGDA‐nSi precursor. E) Expected maximum shear rate as a function of volumetric injection rate, through a typical neurosurgical microcatheter whose diameter is ≈400 microns. Green highlighted region represents typical or recommended injection rate of clinically available neurological injectable embolic devices. Referencing to (A), the expected effective viscosities are in the range of 10^−1^ Pa s during injection.

The hydrogel precursor exhibited a nearly constant decade‐by‐decade reduction in viscosity across a wide range of shear rates ($\dot{\gamma }=\;$0.005–1000 s^−1^) (**Figure** **2**A). This indicates that addition of nSi improves post extrusion stability while maintaining high injectability by imparting shear thinning. The precursor falls within linear viscoelastic regime over a moderate range of strains (Figure 2B), exhibiting the material's ability to maintain a solid‐like state. The step‐shear measurement, cycling between low ($\dot{\gamma }=\;$0.1 s^−1^) and high ($\dot{\gamma }=\;$1000 s^−1^) shear rates several times, has revealed that the nSi‐polymer interaction is highly reversible, allowing the hydrogel to repeatedly break and heal, with the recovery taking place within several seconds (Figure 2C). The step‐strain measurement further demonstrates this solid–fluid reversibility under low (0.1%) and high (100%) strains, wherein the storage modulus (*G*′) is dominant at low strain and the loss modulus (*G*″) becomes dominant at high strain (Figure 2D). Once again, the transition is rapid in both directions and is complete in several seconds. When undergoing injections across a 1.7 French neurological microcatheter (0.15–0.35 mL min^−1^, lumen diameter ≈400 µm), we expect maximum shear rates in the range of 400 to 800 s^−1^ (Figure 2E). Thus, the precursor should effectively reduce in viscosity by approximately three orders of magnitude during injection compared to rest state. The rheological properties of the PEGDA‐nSi gel are such that it is sufficiently injectable over this range of shear rate, exhibiting effective viscosities in the 10^−1^ Pa s order, which are comparable to clinically available liquid embolics like Onyx, whose viscosity options are widely offered between 18 to 500 Pa s.^[^ ^46^, ^47^ ^]^

Compressive cyclic strain test on the crosslinked PEGDA‐nSi hydrogel was also performed at 0.1 Hz at 10% strain. The Young's modulus of the crosslinked PEGDA‐nSi gel was determined to be 319.4 kPa (see Figure S1, Supporting Information). Break strains were determined both compressively by applying 1.5% s^−1^ and −2% s^−1^ strain until fracture, yielding 81.0% and 63.1% break strains respectively (see Figure S2, Supporting Information). The swelling tests of crosslinked PEGDA‐nSi hydrogels were performed both in deionized water and saline, where it exhibited 232.7 ± 17.1% and 139.3 ± 2.07%, respectively. These values fall between the results seen in samples made entirely out of the short chain PEGDA and long chain PEGDA (see Table S2, Supporting Information).

### 2.3 Photomodulated Extrusion Using a Custom Waveguide‐Integrated Microcatheter

**Figure 3:**
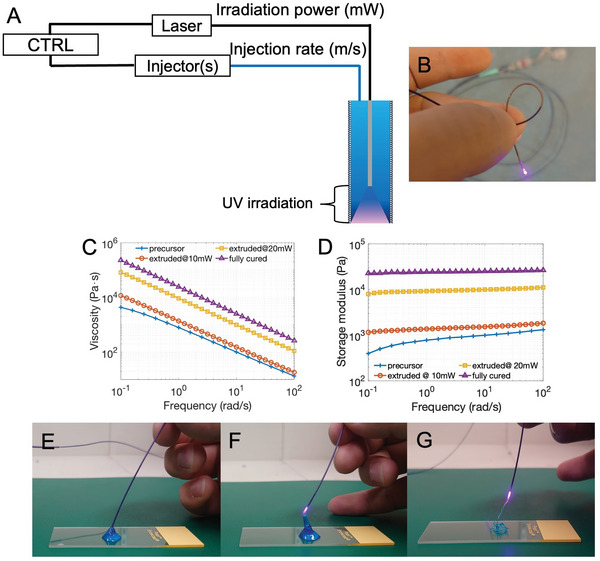
Dynamic photomodulation of PEGDA‐nSi gels. A) A schematic representation of the custom delivery system, comprising of controller unit, UV laser, injector, and optical fiber integrated microcatheters. B) A photograph of the UV emitting microcatheter, which is miniaturized and remains flexible in order to access distal and complex vasculature. C) Viscosity and D) storage modulus as a function of frequency at various irradiation powers, both exhibiting gradual increase with increasing applied optical power. E–G) Photographic demonstrations of in situ modulation of effective extruded viscosities at 0, 10, and 20 mW—corresponding to shear‐recovered semisolid, partially crosslinked and viscosified semisolid, and further solidified hydrogel strings, respectively.

We sought to highlight the versatility of PEGDA‐nSi hydrogel by developing a delivery system capable of leveraging the high injectability of the shear‐thinning precursor and the toughness of the crosslinked hydrogel. We assembled a custom optical fiber integrated microcatheter by retrofitting a 100 µm core multimode optical fiber coaxially across a commercially available microcatheter whose inner diameter was ≈400 µm. The fiber tip was recessed by 5 mm from to the distal port, allowing polymerization immediately prior to and following extrusion (**Figure** **3**A). A 100 mW, 405 nm fiber‐coupled laser source was used with transistor‐transistor logic (TTL) modulation at 10 kHz, enabling real‐time modulation of the optical power, which was suitably swept from 0 to 40 mW during use. The overall catheter assembly was found to maintain adequate bendability and compliance (Figure 3B) to allow for navigation into complex vasculature.

The proposed catheter system enables dynamic tuning of the viscoelasticity of the hydrogel during embolization by modulating the degree of crosslinking_,_ via the modulated optical power of the laser source_._ The degree of crosslinking (*x*_n_) can be dynamically modulated via the optical power (*P*) and/or extrusion velocity (*v*_f_), enabling deposition of hydrogels with varying degrees of viscoelastic properties (*G*). As a demonstration, a flow rate of 0.15 mL min^−1^ was used to extrude the PEGDA‐nSi hydrogel through this custom microcatheter (corresponding to *v*_f_ ≈ 19 mm s^−1^ near the catheter tip) while the optical power emitted at the fiber tip was swept from 0 to 20 mW. Increase in the hydrogel's viscosity (Figure 3C) and storage modulus (Figure 3D) was observed as a function of delivered optical power across all measured shear rates and frequencies.

The high injectability, as well as the ability to photomodulate the viscoelastic properties of the precursor are further shown in photographs of the precursor being injected from the microcatheter at 0, 10, and 20 mW (Figure 3E–G). It is seen that due to the shear recovery of the hydrogel upon exiting the catheter, a solid‐like state is attained even in the absence of UV (Figure 3E). At 10 mW irradiation, the extruded hydrogel exhibits a further increase in modulus, as can be seen by the deposited structure stacking taller (Figure 3F). Consistent with the shear modulus result showing a larger increase at 20 mW, a crosslinked solid hydrogel string is formed within the catheter and subsequently extruded from the catheter (Figure 3G).

### 2.4 Photomodulated Extrusion of Structured Hydrogels for Therapeutic Delivery

**Figure 4:**
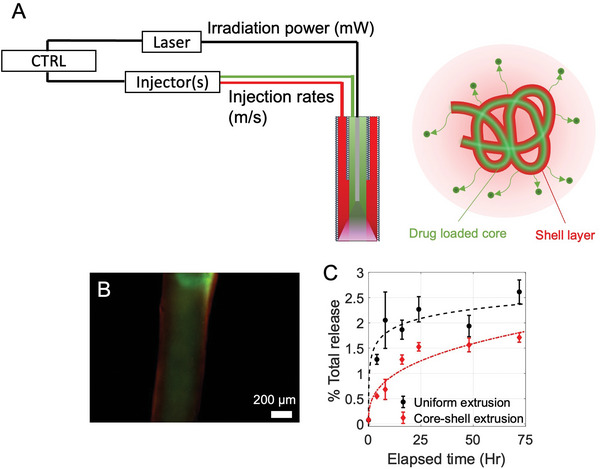
Structured photoextrusion of PEGDA‐nSi gels. A) A schematic representation of the custom delivery system, comprising of controller unit, UV laser, injectors, and optical fiber integrated microcatheters in dual‐lumen configuration. B) Fluorescent micrograph of a core–shell structure extruded by using a dual lumen configuration. The shell was dyed with Rhodamine B, while the core was dyed with FITC‐dextran. C) Cumulative doxorubicin release profile for uniform extrusion (black) and core–shell extrusion (red), showing a more sustained release profile in the core–shell case. Error bars represent 1SD.

To further highlight the capabilities of photomodulated extrusion, we performed multimaterial injection by coaxially arranging two catheters whose inner diameters were ≈900 and 400 µm, with the inner lumen terminating 5 mm proximal to the outer lumen. (**Figure** **4**A). Two PEGDA‐nSi precursors were each dyed with (core) and rhodamine B (shell) in order to differentiate the extrusions from each lumen. The optical fiber was inserted across the inner lumen, with the tip positioned at the midpoint (i.e., 2.5 mm) between the inner and outer lumen tips. An incident power of 20 mW was used, while the flow rates were set to 0.1 and 0.15 mL min^−1^ for inner and outer lumens, respectively. We observed that the extruded hydrogel string resulted in a relatively consistent core–shell structure, due to the laminar flow at the distal segment of the catheter where the two materials are co‐flowing (Figure 4B).

It is largely understood that controlled and sustained release is one of the most desirable characteristics of hydrogel‐based therapeutic delivery vehicles, leading to overall more efficient uptake of the therapeutic by the target tissue.^[^ ^48^, ^49^ ^]^ To demonstrate facile and tunable release, the hydrogel was loaded with doxorubicin, a common anticancer drug which can be used in conjunction with embolization of hypervascular tumors. Doxorubicin was loaded into the PEGDA‐nSi at 100 µg mL^−1^ concentration simply by mixing the prescribed amount to the precursor, which is a similar concentration to previously reported doxorubicin delivery hydrogels.^[^ ^50^, ^51^ ^]^ The doxorubicin is expected to electrostatically interact with nSi, enhancing the overall loading efficiency.^[^ ^50^ ^]^ nSi and doxorubicin was simultaneously introduced to the stock PEGDA solution, after which the mixture was continuously vortexed for at least 1 day. Optical clarity of the mixture was confirmed, which indicated a homogeneous distribution of nSi and its thorough interactions with doxorubicin. The doxorubicin‐loaded PEGDA‐nSi was then extruded in either a single uniform extrusion or a core–shell extrusion where only the core component contained doxorubicin. The extruded samples (200 mg) were each immersed in 1 mL of phosphate buffer saline and the doxorubicin was allowed to diffuse for 72 h. The release profiles (Figure 4C) make it apparent that the encapsulation is effective in lowering the initial rate of release in a core–shell structure, resulting in the initial release profile lasting about three times longer than the uniform sample. The doxorubicin concentration reached a plateau within margins of error in 8 h in the case of uniform extrusion. The concentration continued to increase at a steady for 24 h, followed by a slower increase, in the case of core–shell extrusion. The high retention of the doxorubicin at the endpoint indicates that the buffer was nearing equilibrium with respect to the gels. Previously proposed gels and other carriers making use of electrostatic interactions,^[^ ^52^ ^]^ peptide interactions,^[^ ^53^ ^]^ and self‐assembly^[^ ^54^ ^]^ as their retention mechanisms have demonstrated sustained release durations in a similar time window.

### 2.5 Physical Models of Photomodulated Extrusion and Variable Endovascular Occlusive Capacity

To better understand the photomodulated extrusion process, an analytical model was developed (see section S3, Supporting Information). Assuming steady state crosslinking, the reaction kinetics within the reaction chamber can be represented as^[^ ^55^ ^]^

(1) $$-\frac{\partial \left[M\right]}{\partial t}≅R_{p}=k_{p}\left[M\right]{\left(\frac{\varphi \varepsilon \left[PI\right]I_{z}}{k_{t}}\right)}^{\frac{1}{2}}$$

where [*M*] is the monomer concentration, *R*_p_ is the rate of propagation, *k*_p_ is the propagation constant, *k*_t_ is the termination constant, *φ* is the quantum efficiency of the photoinitiator, *ε* is the extinction coefficient of the photoinitiator, [PI] is the photoinitiator concentration, and *I_z_* is the light intensity at depth *z*. Steady state approximation is appropriate as the forward process will dominate, and we do not expect that the reaction to be diffusion limited. For a precursor flowing at a constant velocity (*v*_f_) in *z*‐direction, the local irradiation intensity is governed by Beer–Lambert's law (*I_z_*) until the infinitesimal precursor volume reaches the light penetration depth $(D_{p}=\;\frac{1}{\ln(10)\varepsilon [PI]}$). Therefore, the resulting degree of polymerization, *x*_n_, can be obtained by integrating the above reaction kinetics over the penetration depth as well as time (see Supporting Information), yielding:

(2) $$\;x_{n}=\;\exp\left[\frac{0.39k_{p}{\left(\varphi I_{o}\right)}^{\frac{1}{2}}}{v_{f}{\left(\varepsilon \left[PI\right]k_{t}\right)}^{\frac{1}{2}}}\right]$$

This relationship can be used to suitably adjust the extrusion parameters to meet a desired degree of crosslinking. Doubling the injection rate, for instance, would in theory require a fourfold increase in the incident light intensity to maintain an equivalent degree of crosslinking. Using a molar extinction coefficient of 2.2 × 10^4^ m ^−1^ m^−1[^ ^56^ ^]^ and initiator concentration of 5 mm, for example, the expected depth of penetration is ≈4 mm, which is similar to the length scale of the reaction chamber used in our proposed catheter. This is such that premature gelation and clogged catheter or, conversely, release of uncrosslinked materials, are prevented.

**Figure 5:**
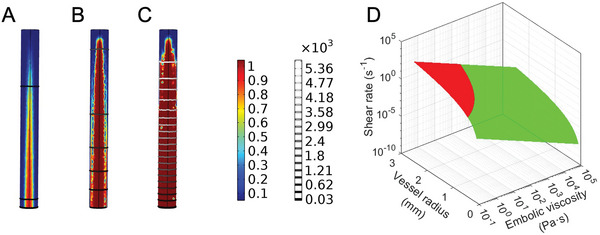
Simulation of viscosity behavior at the catheter tip and prediction of embolization success. A–C) *t* = 10, 30, and 300 ms in a finite‐element simulation of the crosslinking photokinetics, coupled with precursor flow at the catheter tip. Surface heatmap represents the viscosity (scale bar in the units of Pa s), while the contours represent the pressure (scale bar in the units of Pa). Note that due to the constant flow, the distal region within the reaction chamber begins to crosslink, but eventually reaches a steady state where most of the reaction chamber is filled with uniform viscosity precursor. Steady state is reached rapidly (300 ms), allowing precise operational control during embolization. D) Expected local shear rate at the site of embolic deposition, as a function of vessel diameter and embolic viscosity. Light green region indicates sufficient flow stagnation and successful embolization. Red region represents unsuccessful embolization. Here, a resulting local shear rate of 5 s^−1^ was assumed to be sufficient such that the site is eventually thrombosed and healed.

Furthermore, we developed a finite element model by coupling the photokinetics partial differential equations with the Gaussian light distribution as well as fluid dynamics in a geometry representing the reaction chamber. **Figure** **5**A–C (see also section S4 and Video S2, Supporting Information) shows the viscosity as a function of position at various timepoints for a given flow velocity and precursor optical properties. The degree of crosslinking was computed frame‐by‐frame using the photokinetics and optical transmission presented above while the corresponding viscosity was scaled to empirical range of measured viscosity. Using the dynamic viscosity values, the spatial pressure distribution within reaction chamber is also simulated as a function of time and overlaid. As shown in the model, a steady state of precursor flow is attained quickly (≈300 ms), reaching consistent viscosity being extruded at the port. This response time agrees, in which it is possible to rapidly select and change the effective injected viscosities on‐the‐fly. It is also important to ensure a sufficiently low pressure at the tip of the catheter to maintain a smooth continuous flow of the catheter without clogging and risking bursting. The example case being simulated herein indicates a total pressure drop of ≈5 kPa across the reaction chamber, or ≈1 PSI, staying well below the burst pressure of typical injection catheters (200–400 PSI).

Figure 5D is presented to highlight the relationship between an optimal delivery viscosity and its corresponding vessel size (see also section S5, Supporting Information). The model employs Hagen–Poiseuille equation under a simple cylindrical geometry and assumes that a stagnated local shear rate of 5 s^−1^ post embolization will result in thrombosis and hemostasis.^[^ ^57^ ^]^ For an embolic injection of 1 mL, the model segregates the combination of effective injected viscosity and vessel size as successful or unsuccessful scenarios. Here, the range of vessels sizes is classified into large (>4 mm diameter), medium (1.5–4 mm diameter), and small (<1.5 mm diameter)—for which corresponding arterial pressures of 80, 50, and 20 mmHg were assumed as likely.^[^ ^58^ ^]^ The green and red regions predict successful and unsuccessful embolization, respectively. Notably, the model predicts that an effective viscosity of 0.1 Pa s as the precursor is released from the catheter port in the absence of UV would be sufficient to embolize vessels with radii of up to 0.47 mm. On the other hand, an effective viscosity of 10^2^ Pa s would address the entire range of vessels simulated, which is within what is achieved through simple shear recovery of the PEGDA‐nSi interactions alone. However, in the presence of hemodynamic shear and turbulent forces, an additional solidification mechanism such as the proposed photomodulation is warranted.

### 2.6 In Vitro Biocompatibility Assessment of PEGDA‐nSi Hydrogels

**Figure 6:**
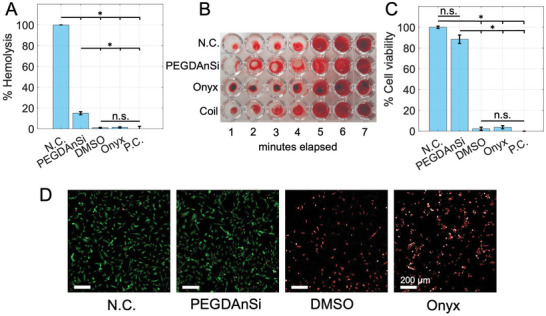
Biocompatibility of PEGDA‐nSi hydrogel. A) Relative hemolysis of red blood cells in contact for 24 h with PEGDA‐nSi compared to Positive Control (deionized water), DMSO, Onyx, and Negative Control (saline), showing low hemolysis potential of PEGDA‐nSi, though higher than Onyx or DMSO. B) Thrombogenicity of whole blood in 96‐well plate in contact with PEGDA‐nSi compared to Negative Control (blank), Onyx, and bare platinum metallic coils, showing decreased clotting time to 3–5 min for PEGDA‐nSi, Onyx, or coils compared to 5–6 min for N.C. C) Relative cell viability of human umbilical vein endothelial cells with indirect contact for 72 h to PEGDA‐nSi compared to Negative Control (blank), DMSO, Onyx, and Positive Control (ethanol), showing much higher cytocompatibilty of PEGDA‐nSi compared to DMSO or Onyx. D) Fluorescent microscopic images (merged live [green] and dead [red]) of Live/Dead Assay of cells after indirect contact for 72 h with Negative Control (blank), PEGDA‐nSi, Onyx, or DMSO, again showing the high cytocompatibility of PEGDA‐nSi compared to other agents. P.C. = positive control, N.C. = negative control. Scale bar = 200 um. Error bars represent 1 SD. *p < 0.05.

PEGDA‐nSi was found to be non‐cytotoxic, hemocompatible, and minimally thrombogenic as shown in **Figure** **6**A,C,D. Cytotoxicity was determined using live/dead assay of human umbilical vein endothelial cells (HUVECs) following indirect contact with PEGDA‐nSi extract (see also Figure S6, Supporting Information). Relative mean cell viability was found to be 88.39 +/− 4.10% for PEGDA‐nSi compared to 3.78 +/− 1.51% for Onyx (*p* = 0.0002). Hemolysis values were higher for the hydrogel compared to Onyx (15.03 +/− 1.48 vs 1.47 +/− 0.50, *p* = 0.0018) but were comparable to other investigational embolization agents.^[^ ^59^ ^]^ In vitro assessment of thrombogenicity showed that clotting times were decreased to 3–5 min when whole blood was in contact with coils, Onyx, or hydrogel, compared to 5–6 min for negative control (blank) as shown in Figure 6B. These findings of biocompatibility were expected due to the inert material selection that is largely orthogonal to biological activities—and thus provides a facile avenue for additional therapeutics to be co‐injected. Our findings of cytotoxicity, thrombogenicity, but low hemolysis potential of Onyx are similar to previously reported values.^[^ ^60^ ^]^

### 2.7 Porcine Preclinical Model Embolization

**Figure 7:**
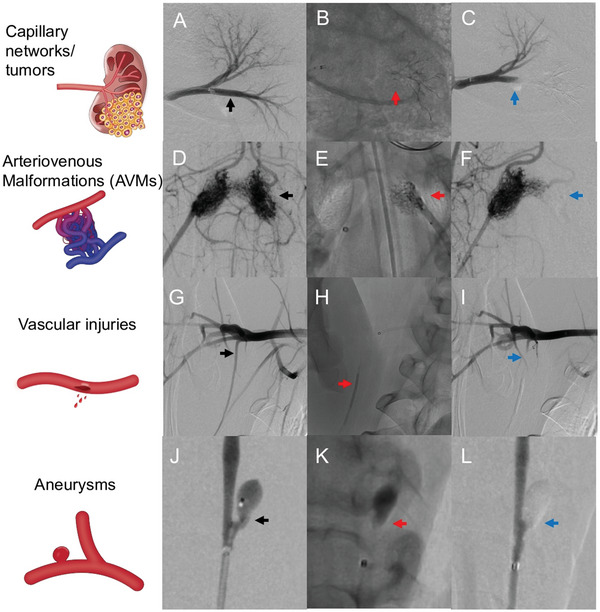
Embolization of vascular targets in a porcine model. After the pre‐embolization angiography (left column: A, D, G, and J), the injected contrast media is allowed to wash out. Immediately following embolization, a single scan of live fluoroscopy (middle column: B, E, H, and K) is taken to visualize the radio‐opaque PEGDA‐nSi (mixed with Omnipaque 300) that has been deposited. Post‐embolization angiography (right column: 7C, F, I, L) is taken to confirm the lack of patency in the treated regions. A) Pre‐embolization angiography of the left renal artery showing the targeted inferior polar artery (black arrow), B) fluoroscopy of PEGDA‐nSi embolization of the inferior polar artery using minimal irradiation for distal penetration into the small arterioles (red arrow), and C) post‐embolization angiography showing obliteration of the inferior polar arterial tree (blue arrow). D) Pre‐embolization angiography of the right ascending pharyngeal artery showing bilateral rete mirabile, E) fluoroscopy of PEGDA‐nSi embolization of the left rete mirabile nidus using moderate light irradiation to fill the nidus without transit into the downstream intracranial vessels or contralateral rete mirabile, and F) post‐embolization angiography showing obliteration of the targeted left nidus (blue arrow) with downstream vasculature remaining patent. G) Pre‐embolization angiography of the right subclavian artery showing the targeted internal thoracic artery (black arrow), H) fluoroscopy of PEGDA‐nSi embolization using high light irradiation to create a solid embolic plug on the internal thoracic artery (red arrow), and I) post‐embolization angiography showing obliteration of the vascular target (blue arrow). J) Pre‐embolization angiography of the left common carotid artery following creation of a venous pouch aneurysm (black arrow), K) fluoroscopy of PEGDA‐nSi balloon‐assisted embolization of the aneurysm (red arrow), and L) post‐embolization angiography showing complete filling of the aneurysm (blue arrow) and patent parent vessel.

Porcine model was chosen for its ability to provide a wide array of vasculatures with high relevance to human anatomy. The aim of this preclinical evaluation was to demonstrate the feasibility of addressing various vascular morphologies by dynamically modulating the viscosity (**Figure** **7**). Several vascular targets were identified to represent vascular or oncologic disease models suitable for embolization. Injection of PEGDA‐nSi was successfully performed in renal capillary beds (Figure 7A–C, see also Video S4, Supporting Information), rete mirabile (Figure 7D–F, see also Video S3, Supporting Information), internal thoracic artery (Figure 7G–I), and wide‐necked aneurysms (Figure 7J–L, see also Video S5, Supporting Information). In this experiment, the PEGDA‐nSi contains iohexol to achieve radio‐opacity. After the pre‐embolization angiography (left column: A, D, G, J), the injected contrast media is allowed to wash out. Immediately following embolization, we take a single scan of live fluoroscopy (middle column: B, E, H, and K) to show where the radio‐opaque PEGDA‐nSi has been deposited. Finally, we take a post‐embolization angiography (right column: C, F, I, L) to confirm the lack of patency in the treated regions. The renal capillary bed has commonly been utilized as an animal model for hepatocellular carcinomas.^[^ ^61^, ^62^ ^]^ The small arterial–arterial network of the rete mirabile is commonly utilized as an animal model for AVMs due to its similarity to a human AVM nidus.^[^ ^63^, ^64^ ^]^ In tumors, distal penetration into tumor vessels is required for devascularization.^[^ ^65^, ^66^ ^]^ In AVMs, on the other hand, complete obliteration of the nidus is required for cure, but premature obliteration of the draining vein(s) of the nidus can lead to AVM rupture and hemorrhage, requiring a fine control of injection.^[^ ^67^, ^68^ ^]^ In all cases, PEGDA‐nSi was injected by hand, keeping with ≈0.2 to 0.3 mL min^−1^ injection rate, which is similar to the recommended injection rate of Onyx. Depending on the vessel size, the irradiation power and timing were suitably switched. For instance, the renal tree was embolized by first injecting PEGDA‐nSi without UV irradiation. We initially observe the gel casting the distal segment of the arterial tree, and gradually filling proximally toward the catheter. When the injection reaches the main inferior renal artery branch, the UV laser is switched on to deposit at a higher viscosity and complete the solidification proximally at 20 mW output power, given the larger vessel diameter and correspondingly a higher blood flow. In the rete mirabile, the injection began with 10 mW irradiation to account for the risk of contralateral penetration by viscosifying the precursor in situ. As the cast of gel formed to the catheter tip in the ascending pharyngeal artery, the power was gradually increased to a maximum of 40 mW, once again fully curing the proximal segment that is immediately distal to the catheter tip. 20 mW of irradiation during injection was found to successfully allow the viscosified precursor to cast the subclavian artery. In wide‐necked aneurysms created via micro‐anastomosis of a venous pouch onto the common carotid artery (see also Figure S7, Supporting Information),^[^ ^69^ ^]^ balloon assisted hydrogel embolization was performed. The injection catheter was placed within the aneurysmal sac and was jailed by the inflated balloon during injection. PEGDA‐nSi was injected at 10 mW irradiation to fill the aneurysm completely, and the optical power was subsequently increased to 40 mW to complete the solidification for an additional 3 min. The crosslinked gel was found to be stable within the aneurysmal sac without prolapsing into the carotid artery nor any undesirable adhesion to the catheter tip, and the follow‐up angiography revealed that the aneurysm was completely obliterated with negligible neck remnant. Furthermore, another angiogram (see Figure S8, Supporting Information) of the common carotid artery showed patency of the external carotid artery branches and the rete mirabile and downstream intracranial vessels, indicating no off target events.

## 3 Discussion

Injectable hydrogels offer promising tools to address the limitations in the current endovascular embolization and therapeutic delivery. Compared to tissue injection, endovascular delivery of hydrogels is particularly challenging due to insufficient localization in the presence of blood flow, complex hydrogel designs to balance the gelation kinetics and mechanical integrity, as well as complex therapeutic loading processes. Finite‐time processes such as ionic crosslinking and Michael type additions present challenges in aligning the gelation with unpredictable procedural workflow and local hemodynamics. Shear thinning hydrogels based on guest–host interactions and polymer to nanoparticle interactions are viable alternatives to address the gelation time dependence.^[^ ^59^ ^]^ However, in many cases the degree of shear thinning may not be sufficient to simultaneously attain high injectability and endovascular stability in distal high‐flow cases.

In this work, we developed a shear‐thinning and photocrosslinkable PEGDA‐nSi hydrogel with a facile synthesis process. Thanks to the reversible polymer‐nSi interactions, the precursor was found to undergo viscosity reduction by approximately three orders of magnitude highly injectable through a 1.7 Fr microcatheter while the strength of the crosslinked hydrogel observed to be excellent. The range of arterial pressures expected, up to 120 mmHg or ≈16 kPa, is well below the strength of the gel, whose compressive modulus is in the order of 100's of kPa with the break strain being well over 50%. Thus, cohesive failure is unlikely once crosslinked, limitation in the maximum blocking force is largely a function of how well the mass of hydrogel is localized within the artery. Swelling is expected to be limited by the inward constrictive forces exerted by the vessel wall and reaches an equilibrium, aiding further to stabilize the gel over time. Given that vessel diameters regularly change due to pulsation and circulatory demands, a free gravimetric swelling ratio of 139.3 ± 2.07% observed in the PEGDA‐nSi is not expected to be a significant concern.

We coupled this PEGDA‐nSi hydrogel with an intracatheter photomodulated extrusion device to combine the benefits of shear thinning/recovery with a highly controllable photosolidification process. Using dynamic photomodulation, we empirically showed the extrusion of hydrogel precursors with viscosities varying by over an order of magnitude by way of modulating the incident UV power for a given precursor flow rate. The theory of solidification control was developed by coupling the photokinetics of crosslinking reaction with Beer–Lambert law as well as the flow velocity across the reaction chamber. Particularly in distal vascular embolization where clinically available options are plagued by deployment control issues such as off target embolics and glued catheters, the proposed approach offers a significant improvement.

We further showed that intracatheter photomodulated extrusion could be expanded to offer further capabilities such as facile and controlled therapeutic delivery. By employing a dual‐lumen catheter while similarly integrating the optical waveguide, we demonstrated coaxial extrusion of structured hydrogels and strategically load and encapsulate doxorubicin for controllably sustained release. By modifying the lumen designs and corresponding flow profiles in the catheter, it is in theory possible to further optimize the release profile. Likely, a custom single‐piece extrusion incorporating the two lumens is necessary for practical use as the method used herein to coaxially assemble two commercially available catheters results in a device that is too stiff. Loading techniques in previously proposed injectable hydrogels often require grafting the drug molecules onto specifically engineered polymers^[^ ^54^ ^]^ or nanoparticles,^[^ ^70^, ^71^ ^]^ or encapsulating in self assembling hydrogel nanoparticles^[^ ^72^ ^]^ for sustained or differential release. These approaches are not only complex but can be selective to the therapeutics being delivered. The ability to simply mix the therapeutics into a precursor and macroscopically encapsulate the hydrogel may be an avenue toward widespread use of hydrogel‐based drug delivery in endovascular contexts.

Last, we demonstrated a practical utilization of intracatheter photomodulated extrusion in a porcine model. Whereas, clinicians currently strategically pre‐select an appropriate agent from a wide variety of embolics available based on the expected hemodynamics, our system enables adapting a given embolic formulation to suit a wide range of cases.^[^ ^73^, ^74^ ^]^ Often the preprocedural estimation of hemodynamics, based on digital subtraction angiography (DSA) of the diseased vessels minutes prior to embolic injection, can be inaccurate due to a number of factors such as inexperience and vessel spasms, resulting in suboptimal embolization, off‐target embolic release, and iatrogenic morbidities. Intracatheter photomodulation addresses this critical gap, allowing the operator to observe the real‐time fluoroscopic feedback and adjust the embolic properties accordingly. Namely, in filling smaller vessels in the range of 1 mm or less in diameter, the effective precursor viscosity as it exits the catheter port (≈0.1 Pa s), which rapidly increases once released into blood vessels, is predicted to be sufficient to stagnate the flow, and thus in practice, renal trees were embolized by first allowing the precursor to free flow distally without any photoirradiation. This eventually occluded the terminal branches and backflowed toward the catheter tip, at which point the UV irradiation was performed for a suitable period of time. On the other hand, in embolizing the subclavian artery which is more proximal to the heart and is typically sized between 2 and 3 mm in diameter, viscosification of the precursor to hundreds of Pascal‐seconds was predicted to be required for a successful embolization. In practice, the laser was set to 20 mW during injection, corresponding to an expected viscosity of over 100 Pa s at shear rates below 10 rad s^−1^, which proved to successfully embolize the structure. Overall, it is envisaged that this predictability of photomodulated extrusion approach will provide a more reliable and safe clinical experience in usage of liquid embolics. This preclinical evaluation was limited by the acute nature of the animal models, and further trials are necessary to confirm long term systemic effects of hydrogel embolization to supplement current findings of in vitro biocompatibility.

## 4 Conclusion

Unlike tissue injection, endovascular delivery of hydrogels presents unique challenges due to unpredictable local hemodynamics, combined with the injectability and gelation needs across low‐profile catheters. In this work, we presented a hydrogel design to simultaneously utilize shear‐thinning and photocrosslinking to achieve a high injectability and a greater degree of control over the solidification process, while keeping with simple constituents and facile synthesis. The reversible interactions between PEGDA and nSi enabled injection through a clinically relevant microcatheter with ease. The crosslinked gel was shown to be adequately resistant to both strain and swelling. Combined with dynamic photomodulated microcatheter injection, the hydrogel precursor could readily be delivered, localized, and hemostatic across a great range of vascular morphologies and hemodynamics in porcine. Furthermore, photomodulated structured extrusion was capable of encapsulating doxorubicin to achieve a more sustained release compared to unencapsulated payload. This work has addressed the current limitations in endovascular embolization and further proposed a scalable platform for endovascular therapeutic delivery.

## 5 Experimental Section

### Synthesis of PEGDA‐nSi Hydrogels

PEGDA (*M*_w_ = 700, Aldrich 455 008), PEGDA (*M*_w_ = 10k, PolySciences 26 279), Lapointe XLG (BLK), and lithium phenyl‐2,4,6‐trimethylbenzoylphosphinate (LAP, Aldrich, 900 889) were purchased and used without further modifications or purifications. nSi was first suspended in deionized water and vigorously vortexed until homogeneous and translucent. The mixture was then allowed to rest still until the color became transparent, indicating full hydration of the silicate particles (up to 1 day). Then, a mixture of PEGDA, as well as LAP was added into the nSi‐water mixture and further thoroughly mixed. The final mixture was thoroughly degassed and stored in dark at room temperature until use. To make precursors used for porcine models, the same procedure was followed but fully replacing the deionized water with a radiopaque contrast agent (Omipaque 300, GE Healthcare). See Table S1, Supporting Information, which summarizes the several versions of the PEGDA‐nSi compositions. Both of the nSi2 variants were found to be compatible with the 1.7 Fr microcatheter, while both of the nSi4 variants were compatible with the 2.7 Fr microcatheter. Unless otherwise noted, PEGDA‐nSi in the text refers to PEGDA‐nSi2‐b (*b stands for blended).

### Optical Fiber Integrated Microcatheters and Control System

A custom optical fiber integrated microcatheter was assembled by retrofitting a 100 µm core, 0.22 NA multimode optical fiber (AFM100H, Thorlabs, Newton, New Jersey) coaxially across a commercially available 1.7 or 2.7 French microcatheters (Excelsior SL10/XT‐27, Stryker, Kalamazoo, MI). The fiber was terminated using an SMA connector on the proximal end, while the distal end was flat cleaved. The fiber tip was positioned 5 mm from to the distal tip of the catheter lumen, leaving the gap distance in which the precursor flow takes places in the presence of UV, and fixated using a rotating hemostatic valve at the proximal end of the catheter. The overall catheter assembly maintained adequate bendability and compliance (see Figure 1 photograph) to allow for navigation into complex vasculature.

A 100 mW, 405 nm fiber‐coupled laser source (CNI laser, Changchun, China) was used with TTL modulation at 10 kHz enabling real‐time modulation of the optical power, which was suitably swept from 0 to 40 mW during use. A DAQ (USB6351, National Instruments) was used to interface with the laser unit via PWM, and the syringe pumps were serially driven from the computer. MATLAB was used to simultaneously communicate with all components simultaneously.

### Demonstration of Structured PEGDA‐nSi Hydrogel Extrusion and Assessment of Controlled Drug Release

To demonstrate that a core–shell hydrogel extrusion from concentrically arranged microcatheters using the proposed photomodulation method, PEGDA‐nSi precursors were mixed with FITC‐dextran (FD500S, Aldrich) and rhodamine B (83 689, Aldrich) each at a concentration of 0.2 mm. A 1.7 (Excelsior SL10) and a 3 (3MAX, Penumbra, California) French microcatheter were coaxially assembled with the inner lumen terminating 5 mm proximal to the outer lumen. The optical fiber was inserted across the inner lumen, with the tip positioned at the midpoint (i.e., 2.5 mm) between the inner and outer lumen tips. An incident power of 20 mW was used, and the flow rate was each set to 0.1 and 0.15 mL min^−1^ for inner and outer lumens, respectively. The extruded structure was then visualized using fluorescence microscopy (Axio Observer, Zeiss).

To demonstrate tunable therapeutic release, doxorubicin, a common chemotherapy drug, was used. Doxorubicin (D1515, Aldrich) was loaded into the PEGDA‐nSi at 100 µg mL^−1^ concentration. nSi and doxorubicin were simultaneously introduced to the stock PEGDA solution, after which the mixture was continuously vortexed for at least 1 day. Optical clarity of the mixture was confirmed, which indicated a homogeneous distribution of nSi and its thorough interactions with doxorubicin. The doxorubicin‐loaded PEGDA‐nSi was then extruded in either a single uniform extrusion or a core–shell extrusion where only the core component contained doxorubicin. 200 mg of extrusion samples were each immersed in 1 mL of PBS, and 30 µL samples were collected at various timepoints for up to 72 h. The fluorescence of doxorubicin (Ex/Em: 470/560) in the buffer at each timepoint was measured using the Synergy Neo2 multimode plate reader (Biotek).

### Swelling Test of PEGDA‐nSi Hydrogels

Swelling test was performed by first completely curing 0.5 g of a PEGDA‐nSi hydrogel precursor under a 405 nm UV lamp (10 mW cm^−^^2^) for 20 min and submerging the cured piece of gel into a 50 mL of either deionized water or saline bath. The gels were left submerged and allowed to swell for a period of 1 week at room temperature. The weights prior to and after the swelling were recorded. Experiment was carried out in triplicates and the standard deviations were calculated.

### Rheological Characterization of PEGDA‐nSi Hydrogels

All characterization was carried out using Discovery HR3 Hybrid Rheometer (TA Instruments). A 1 cm flat plate was used with a gap distance of 500 µm. All measurements were carried out at 25 °C. Shear rate sweep measurement was carried out from 0.005 to 1000 s^−1^. Oscillatory frequency sweep measurements were performed by sweeping between 0.1 and 100 rad s^−1^. For characterization of extruded precursors at varying irradiation power, samples were collected into Eppendorf tubes for while the syringe pump and laser unit were set at a fixed flow rate and optical power, respectively. The tube was then wrapped in aluminum foil to prevent further photocrosslinking and was characterized within a day. Step‐shear measurements were performed at shear rates oscillating between 0.1 and 1000 s^−1^. Step strain measurements were carried out at strains oscillating between 0.1% and 100%. Processing and analyses were performed using TRIOS (TA Instruments) software, or MATLAB.

### Mechanical Characterization of Crosslinked PEGDA‐nSi Hydrogels

A Bose Electroforce 3000 series mechanical analyzer was used to determine the modulus by applying a sinusoidal compression at a frequency of 0.1 Hz with a 12.5% peak strain. For break‐strain tests, a NewPort XPS Q8 Motion Controller in combination with a load cell (LH‐SZ‐02, LH Sensor) were used to drive an indenter at a desired compression speed until fracture was observed via a drop in the load.

### Cytotoxicity Testing

Human umbilical vein endothelial cells (HUVEC; C0035C, ThermoFisher, Waltham, MA) were cultured according to the protocol of the supplier. Hydrogel and Onyx extracts were prepared by incubating 50 μL in 1 mL of cell culture media at 37 °C for 24 h. HUVECs were seeded at 0.2 × 10^4^ per well into a 96‐well plate. Cells were cultured until subconfluency. Extracts of hydrogel and Onyx were prepared by incubating 50 μL of the agent in 1 mL of cell culture media at 37 °C for 24 h, then centrifuging to obtain the supernatant. The cells were dosed with 50 μL of hydrogel extract, 50 μL of DMSO, 50 uL of hydrogel extract, 50 μL of Onyx extract, or left blank (negative control). All experiments were performed in triplicate. After 3 days, cells were stained with calcein AM and ethidium homodimer‐1 utilizing the LIVE/DEAD viability assay (L3224, ThermoFisher, Waltham, MA) according to protocol. The images were collected using a fluorescent camera (Opera Phenix; Perkin Elmer, Waltham, MA) and edited using a cell analysis tool (Harmony 4.9; Perkin Elmer, Waltham, MA). Relative cell viability compared to the negative control was expressed as percentages, with mean and standard deviation. Two‐way *t*‐tests were performed to compare between group means.

### Hemolysis Testing

Diluted human red blood cells were utilized for hemolysis testing via a hemolysis test kit according to the protocol of the supplier (Haemoscan BV, Groningen, Netherlands). Small pieces of hydrogel and Onyx (0.5 cm diameter) were prepared under sterile conditions and submerged in 1.5 mL Eppendorf tubes with 1 mL of diluted red blood cells. Negative controls with normal saline and positive controls with deionized water were also prepared. These samples were incubated at 37 °C with gentle end‐over‐end rotation for 24 h. Samples were then centrifuged at 4000 g for 1 min and the supernatant was transferred into the wells of a 96‐well plate. Tests were performed in triplicate. The hemoglobin concentration of each sample was measured via spectrophotometer (M1000 Infinite Pro; Tecan, Männedorf, Switzerland), at 380, 415, and 450 nm. The optical density was calculated using the Harboe method of (2 × 415) − (450 + 380). Percent hemolysis was calculated via the equation: % hemolysis = (OD_sample_ − OD_neg_/OD_pos_), where OD_sample_ is the optical density of the sample, OD_neg_ is the optical density of negative control, and OD_pos_ is the optical density of the positive control. Values were expressed as mean with standard deviation, and two‐way *t*‐tests were performed to compare between group means.

### Coagulation Testing

Whole porcine blood was utilized for coagulation testing. Small pieces (0.5 cm diameter) of hydrogel, Onyx, and segments of bare platinum metallic coils were placed in a 96‐well plate, alongside wells that were left blank (negative control). 50 μL of untreated whole porcine blood was added to each well. At 1 min intervals, the wells were washed with 50 μL of NS and the liquid was aspirated to leave only coagulated blood. Clotting time was assessed by qualitative analysis of each well.

### Porcine Model Experiments

All experiments in this work were approved by Animal Use Protocol 20724/21724 at Sunnybrook Research Institute. Five Yorkshire swine weighing 40–45 kg were utilized. All procedures were performed under general anesthesia with continuous hemodynamic monitoring. A dedicated animal interventional radiology suite equipped with a single‐plane C‐Arm (Philips, Andover, MA) was used for all procedures. Ultrasound guided right femoral punctures were performed and a 6 French (F) sheath was inserted into the right common femoral artery. A 6F Envoy (Codman, Boston, MA) guide catheter with guidewire was navigated with roadmap technique into the right brachiocephalic artery, left renal artery, or left ascending pharyngeal artery. Pre‐embolization angiograms were performed. An Excelsior XT‐27 catheter (Stryker Kalamazoo, MI) with Transcend micro guidewire (Stryker, Kalamazoo, MI) was then navigated to the site of embolization (internal thoracic artery, inferior polar artery of the kidney, and entrance of the rete mirabile). The micro‐guidewire was then replaced with the optical fiber. The laser intensity was selected and modulated throughout the hydrogel injection process. Embolization of the vascular target was performed under fluoroscopic guidance. The injection catheter was then removed, and post‐embolization angiography is performed to confirm full occlusion of the vessel and assessment of any off‐target embolization in downstream vasculature.

A wide necked aneurysm was created on the left common carotid artery using the venous pouch microanastomosis method.^[^ ^75^ ^]^ Briefly, a 1 cm portion of the left external jugular vein is harvested. The left common carotid artery was isolated at the mid‐cervical region, temporary clips were applied, and a 5 mm arteriotomy was performed on the anterolateral wall. The venous pouch was then connected end‐to‐side to the arteriotomy with running 8‐0 Prolene sutures (Ethicon, Cincinnati, OH). The temporary clips were released. The 6F envoy was navigated to the common carotid artery and an angiogram was performed to confirm patency of the aneurysm and parent vessel. See Figure S1, Supporting Information, for a photograph of the anastomosed venous pouch aneurysm.

The injection catheter (Excelsior SL10; Stryker, Kalamazoo, MI) was navigated into the aneurysm, then the micro guidewire was replaced with the optical fiber. A balloon catheter (TransForm, Stryker, Kalamazoo, MI) was navigated into the carotid artery covering the neck of the aneurysm and inflated. Injection of low‐viscosity hydrogel was performed to fill the aneurysm, then the laser was set to 40 mW to allow for photopolymerization for 3 min. The balloon catheter was then deflated (total time 5 min). The injection catheter and balloon catheters were then removed, and post embolization angiography was performed.

### Statistical Analysis

All in vitro data approximated a normal distribution and are reported as mean ± standard deviation. Pairwise Student *t*‐tests were used for statistical difference between variables with a Bonferroni correction used for multiple comparisons. For all statistical tests, a threshold value of *p* < 0.05 indicated significance.

## Conflict of Interest

The authors declare no conflict of interest.

## Supporting information

Supporting Information

Supplemental video 1

Supplemental video 2

Supplemental video 3

Supplemental video 4

Supplemental video 5

## Data Availability

The data that support the findings of this study are available in the supplementary material of this article.
