# Supplementary material for: Photomodulated Extrusion as a Localized Endovascular Hydrogel Deposition Method
Source: Adv Healthc Mater. 2023 Feb 3;12(12):2202632. doi: 10.1002/adhm.202202632 (PMC11468792; doi:10.1002/adhm.202202632)
Supplement: Supplementary file 1 — Supporting Information [file ADHM-12-2202632-s004.pdf]

ADVANCED HEALTHCARE MATERIALS

Supporting Information

for *Adv. Healthcare Mater.*, DOI 10.1002/adhm.202202632

Photomodulated Extrusion as a Localized Endovascular Hydrogel Deposition Method

Yuta Dobashi, Jerry C. Ku, Joel Ramjist, Christopher Pasarikovski, Konrad Walus, John D. W. Madden and Victor X. D. Yang**

Supplementary Materials for

Photomodulated extrusion as a localized endovascular hydrogel deposition method

Yuta Dobashi[1,2*†] Jerry C. Ku [1,2,3*†], Joel Ramjist [2], Christopher Pasarikovski[1,2,3], Konrad Walus[4], John Madden[4], Victor XD Yang[1,3†]

S1. Summary of compositions of PEGDA-nSi hydrogels

**Formulation**	**PEGDA 700**	**PEGDA 10k**	**nSi**	**LAP**
**PEGDA-nSi2-s**	15 %w/v	N/A	2 %w/v	0.5 %w/v
**PEGDA-nSi4-s**	15 %w/v	N/A	4 %w/v	0.5 %w/v
**PEGDA-nSi2-l**	N/A	15 %w/v	2 %w/v	0.5 %w/v
**PEGDA-nSi2-b**	10 %w/v	5 %w/v	2 %w/v	0.5 %w/v

Table S1. A list of PEGDA-nSi hydrogel compositions. The nSi2 variants were found to be compatible with the 1.7 Fr microcatheter, while the nSi4 variant was only compatible with the 2.7 Fr microcatheter. As the preclinical evaluation required 1.7-Fr catheter compatibility, the nSi2 variant was chosen and optimized for swelling by molecular weight blending. Unless otherwise noted, PEGDA-nSi in the main text refers to PEGDA-nSi2-b (** s stands for short, l stands for long, and b stands for blended).

S2. Mechanical properties of the PEGDA-nSi hydrogels

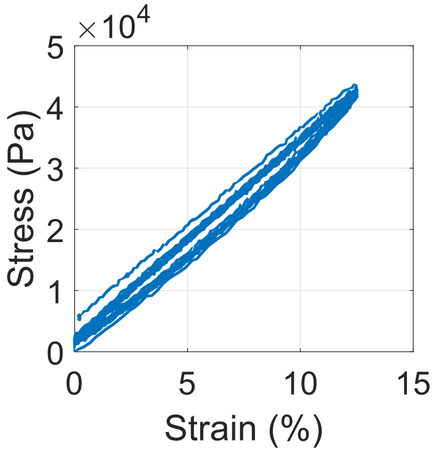

Figure S1. Stress-strain curve of crosslinked PEGDA-nSi gel (cyclic compressive strain of 12.5% at 0.1 Hz). A compressive sinusoidal strain of 12.5% was applied at 0.1 Hz, and the resulting stress was recorded. The stress-strain curve was fitted with a linear slope to give the modulus of 319.4 kPa.

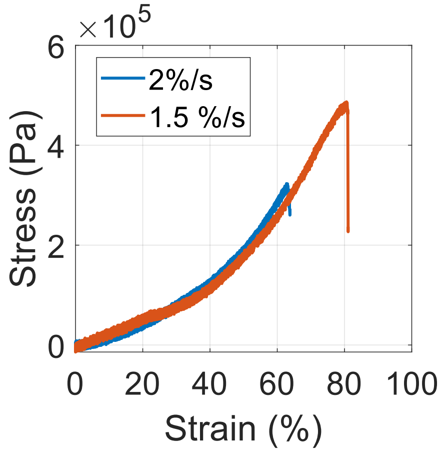

Figure S2. Break strain test of crosslinked PEGDA-nSi gel with 2%/s (blue) and 1.5%/s (orange) constant compressive strains applied, showing the breaks occurring at 63.1 and 81.0% strains respectively.

S2. Swelling properties of the crosslinked PEGDA-nSi hydrogels

**Sample**	**Medium**	**Swelling ratio**
**PEGDA-nSi2-s**	DI water	104.8 ± 0.96 %
**PEGDA-nSi4-s**	DI water	109.6 ± 2.44 %
**PEGDA-nSi2-l**	DI water	268.5 ± 7.96 %
**PEGDA-nSi2-b**	DI water	232.7 ± 17.1 %
**PEGDA-nSi2-s**	Saline	95.3 ± 0.67 %
**PEGDA-nSi4-s**	Saline	96.8 ± 2.12 %
**PEGDA-nSi2-l**	Saline	235.4 ± 2.51 %
**PEGDA-nSi2-b**	Saline	139.3 ± 2.07 %

Table S2. Swelling ratios PEGDA-nSi hydrogel compositions. Swelling test was performed by first completely curing 0.5 grams of a PEGDA-nSi hydrogel precursor under a 405nm UV lamp (10mW/cm2) for 20 minutes and submerging the cured piece of gels into a 50 ml of either deionized water or saline bath. The gels were left submerged and allowed to swell for a period of 1 week at room temperature. The weights prior to and after the swelling were recorded. Experiment was carried out in triplicates and the standard deviations were calculated.

S3. Analytical model of photokinetics within the reaction chamber

The process of photocrosslinking may differ depending not only on the irradiation energy, but also the flow rate and the individual hydrogel precursors. A polymerization and crosslinking reaction comprises of initiation, propagation, and termination steps. Assuming a steady state (i.e. rate of initiation is matched by the rate of termination) condition, the reaction kinetics are described as follows:

$$-\frac{\partial[M]}{\partial t} = \boldsymbol{R_i} + \boldsymbol{R_p} \cong \boldsymbol{R_p} \text{ and} \qquad (1)$$

$$\boldsymbol{R_p} = \boldsymbol{k_p}[\boldsymbol{M}](\frac{\boldsymbol{R_i}}{2\boldsymbol{k_t}})^{\frac{1}{2}}, \text{ where} \qquad (2)$$

$$\boldsymbol{R_i} = 2\boldsymbol{\varphi\epsilon}[\boldsymbol{PI}]\boldsymbol{I_z}. \qquad (3)$$

Here, $[\boldsymbol{M}]$ is the monomer concentration, $\boldsymbol{R_i}$ is the rate of initiation, $\boldsymbol{R_p}$ is the rate of propagation, $\boldsymbol{k_p}$ is the propagation constant, $\boldsymbol{k_t}$ is the termination constant, $\boldsymbol{\varphi}$ is the quantum efficiency of the photoinitiator, $\boldsymbol{\epsilon}$ is the extinction coefficient of the photoinitiator, $[\boldsymbol{PI}]$ is the photoinitiator concentration, and $\boldsymbol{I_z}$ is the light intensity at depth z. Steady state approximation is appropriate as in the polymerization process the forward reaction dominant, and we do not expect any diffusion limited regime. A given volume of precursor flowing at a constant velocity ($\boldsymbol{v_f}$) in z direction will be subject to local intensity according to Beer-Lambert's law ($\boldsymbol{I_z}$) until it the light penetration depth ($\boldsymbol{D_p} = \frac{1}{\ln(10)\epsilon[PI]}$). Thus, the resulting degree of polymerization, $\boldsymbol{x_n}$, can be obtained by modifying the above expression by integrating over the penetration depth as well as time of flow under photo irradiation ($\boldsymbol{D_p}/\boldsymbol{v_f}$). We begin with [2]:

$$\ln\left(\frac{[\boldsymbol{M}]_o}{[\boldsymbol{M}]}\right) = \left[\frac{k_p^2\varphi\varepsilon I_o[PI](10^{-\varepsilon[PI]z})}{k_t}\right]^{\frac{1}{2}} \boldsymbol{t} \qquad (4)$$

where $\frac{[\boldsymbol{M}]_o}{[\boldsymbol{M}]}$ is defined as the degree of polymerization, $\boldsymbol{k_p}$ is the propagation constant, $\boldsymbol{k_t}$ is the termination constant, $\boldsymbol{\varphi}$ is the quantum efficiency of the photoinitiator, $\boldsymbol{\epsilon}$ is the extinction coefficient of the photoinitiator, $[\boldsymbol{PI}]$ is the photoinitiator concentration, and $\boldsymbol{I_z}$ is the light intensity at depth z. To obtain the final degree of crosslinking at the exit port of the catheter, we integrate the expression on the right-hand side as follows:

$$\ln\left(\frac{[\boldsymbol{M}]_o}{[\boldsymbol{M}]_{extruded}}\right) = \int_0^{\frac{D_p}{v_f}}\int_0^{D_p}\left[\frac{k_p^2\varphi\varepsilon I_o[PI](10^{-\varepsilon[PI]z})}{k_t}\right]^{\frac{1}{2}} \boldsymbol{t}\, \boldsymbol{dzdt}$$

$$= \int_0^{\frac{D_p}{v_f}} \frac{2\left[\frac{k_p^2 \varphi \varepsilon I_o [PI]\left(10^{-\varepsilon[PI]z}\right)}{k_t}\right]^{\frac{1}{2}}}{\varepsilon[PI]\ln(10)} t\, dt \Bigg|_0^{D_p}$$

$$= \int_0^{\frac{D_p}{v_f}} \frac{2k_p\left[\varphi I_o\left(10^{-\varepsilon[PI]D_p}\right)\right]^{\frac{1}{2}}}{(\varepsilon[PI]k_t)^{\frac{1}{2}}} t\, dt - \int_0^{\frac{D_p}{v_f}} \frac{2k_p(\varphi I_o)^{\frac{1}{2}}}{(\varepsilon[PI]k_t)^{\frac{1}{2}}} t\, dt$$

$$= \frac{k_p\left[\varphi I_o\left(10^{-\varepsilon[PI]D_p}\right)\right]^{\frac{1}{2}}}{(\varepsilon[PI]k_t)^{\frac{1}{2}}} t^2 \Bigg|_0^{D_p/v_f} - \frac{k_p(\varphi I_o)^{\frac{1}{2}}}{(\varepsilon[PI]k_t)^{\frac{1}{2}}} t^2 \Bigg|_0^{D_p/v_f}$$

$$= \frac{k_p(\varphi I_o)^{\frac{1}{2}}}{v_f(\varepsilon[PI]k_t)^{\frac{1}{2}}}\left(10^{-\frac{1}{2}\varepsilon[PI]D_p} - 1\right)$$

$$\therefore \bar{x}_n = \frac{[M]_o}{[M]_{extruded}} = exp\left[\frac{k_p(\varphi I_o)^{\frac{1}{2}}}{v_f(\varepsilon[PI]k_t)^{\frac{1}{2}}}\left(10^{-\frac{1}{2}\varepsilon[PI]D_p} - 1\right)\right]$$

Using $D_p = \frac{1}{\ln(10)\varepsilon[PI]}$, we obtain:

$$\bar{x}_n = \frac{[M]_o}{[M]_{extruded}} = exp\left[\frac{k_p(\varphi I_o)^{\frac{1}{2}}}{v_f(\varepsilon[PI]k_t)^{\frac{1}{2}}}\left(10^{-\frac{1}{2\ln(10)}} - 1\right)\right]$$

$$= exp\left[\frac{0.39 k_p(\varphi I_o)^{\frac{1}{2}}}{v_f(\varepsilon[PI]k_t)^{\frac{1}{2}}}\right] \qquad (5)$$

S4. Numerical simulation of the photokinetics, viscosities, and the pressures within the reaction chamber

COMSOL MultiPhysics, employing Radiative Beam and Absorbing Media and Laminar Flow modules, was used to perform numerical simulations within the reaction chamber. A cylindrical geometry with a diameter of 400 μm and a length of 5 mm was used as seen below:

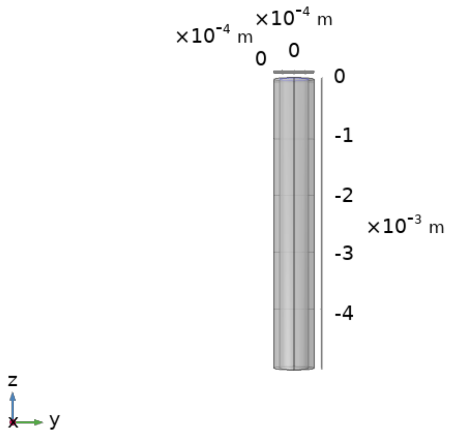

Figure S3. Geometry of the reaction chamber

The incident beam was set to have a power of 10 mW, which was directed in -z direction and assumed to have a Gaussian distribution with a standard deviation of 0.1 mm in order to represent the beam profile of the optical fiber used in the custom catheter setup. Using an extinction coefficient of $7.5 \cdot 10^3$ $M^{-1}m^{-1}$ [3] and initiator concentration of 5 mM, for example, the expected depth of penetration is approximately 11 mm, which is sufficient to cover the length of the reaction chamber used in our proposed catheter, as can be seen in the normalized intensity map below across the reaction chamber showing approximately 10% of initial intensity remaining at the distal end.

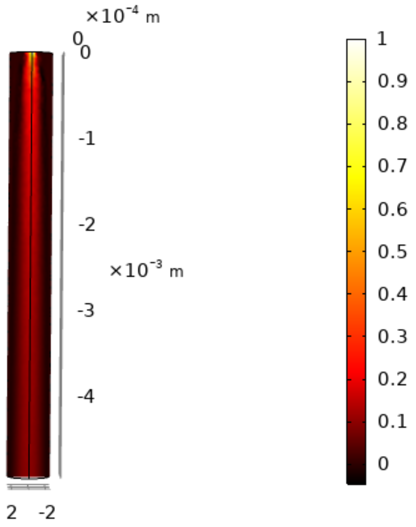

Figure S4. Normalized optical intensity distribution across the reaction chamber

At the inlet, the boundary condition was set in terms of volumetric flow rate, which was 0.15 ml/min. The outlet boundary condition was set to have 0 Pa pressure. No-slip conditions were applied to the wall. The precursor material was set to have a density of 1100kg/m^3, an absorption coefficient determined by the product of initiator concentration and extinction coefficient at 405 nm (other components were assumed to have negligible absorption), and an initial dynamic viscosity of 0.05 Pa·s.

The degree of polymerization was calculated similarly to the analytical approach using the following equation:

$$\bar{x}_n = exp\left\{\left[\frac{{k_p}^2\varepsilon[PI]\varphi I_z}{k_t}\right]^{\frac{1}{2}} t\right\}, \qquad (6)$$

where I_z is numerically computed in the geometry while the time is continuously integrated in order to yield the total optical exposure a given volume of precursor is subject to as it travels down the reaction chamber, and accordingly adjust the viscosity. Below are degree of polymerization maps (log $(\bar{x}_n)$) within the reaction chamber taken at 0ms, 30 ms, and 300 ms, and 500 ms (from left to right) of the simulation.

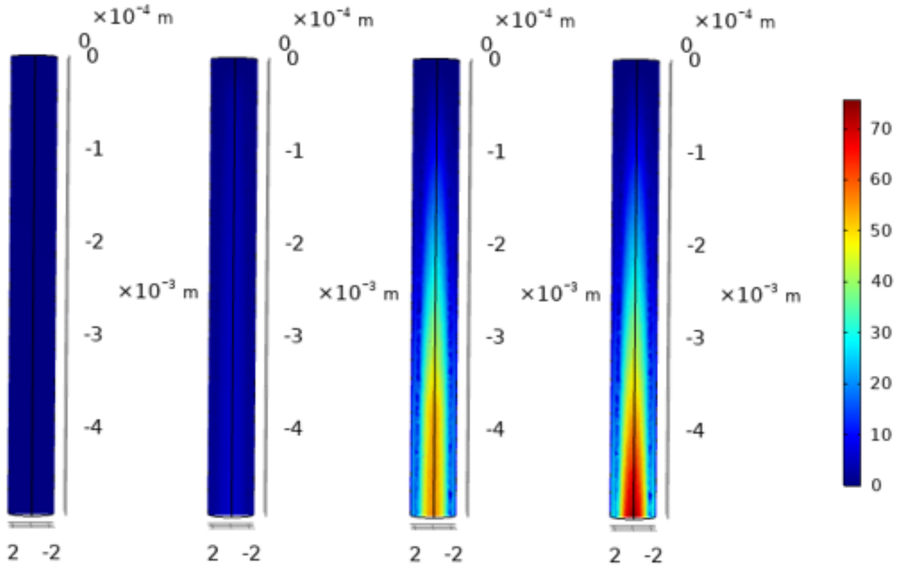

Figure S5. Heatmap representing log $(\bar{x}_n)$ across the reaction chamber at t = 0, 30, 300, 500 ms (from left to right)

The viscosity was fitted with the change in degree of polymerization using upper and lower bounds for viscosity, denoted $\mu_{initial}$ and μ_{final} respectively as follows [4]:

$$\mu = \mu_{\ init} + \frac{\mu_{final}}{1+\exp\left[-\kappa\left(p-p_{half}\right)\right]}, \qquad (7)$$

where κ is a shape control parameter, and $p = 1 - {\bar{x}_n}^{-1}$. The model indicates that the degree of polymerization continues to increase after 300 ms, though in terms of viscosity, stabilized and reached a steady state (see viscosity map in the main text Figure 3 I, J, and K), meaning ${\bar{x}_n}^{-1}$ is negligibly small at 300 ms. Note that with the volumectric flow rate used in the simulation, the

residence time of a given volume of precursor within the reaction chamber is approximately 500 ms.

The table below summarizes the quantities used for the simulation:

**Variable**	**Value**	**Units**
μ_{init}	0.05	Pa·s
μ_{final}	1	Pa·s
Volumetric flow rate	$2.5\cdot10^{-9}$	m^3/s
p_{half}	0.8	N/A
κ	20	N/A
Density of precursor	1100	$kg\cdot m^{-3}$
Catheter wall density	1200	$kg\cdot m^{-3}$
Catheter wall Young's modulus	$1\cdot10^{8}$	Pa
Catheter wall Poisson's ratio	0.499	N/A
Incident optical power	$1\cdot10^{-2}$	W
Photoinitiator concentration	5e-3	M
Extinction coefficient of photoinitiator	$7.5\cdot10^{-3}$	$M^{-1}m^{-1}$
Propagation coefficient	$2\cdot10^{2}$	$M\cdot s^{-1}$
Termination coefficient	$5\cdot10^{6}$	$M\cdot s^{-1}$
Quantum efficiency of photoinitiator	0.1	N/A

Table S3. List of quantities used for the photokinetic and flow simulation

S5. Estimation of required viscosity as a function of vascular geometry and local blood pressures

The aim of this section is to provide an estimate for the required viscosity in order establish hemostasis in various blood vessel sizes and local pressures. To simplify the problem, we consider a simple rigid cylindrical geometry with varying radii as well as pressure gradients.

Using Poiseuille equation, the flow rate of a given blood vessel is

$$q = \frac{\pi r^4}{8\mu L}\Delta P, \qquad (8)$$

where q is the volumetric flow rate, r is the radius of the blood vessel, μ is the dynamic viscosity of the injected gel, L is the length of the embolized segment, ∇P is the pressure drop across the embolized segment. The shear rate in a simple cylindrical geometry is given by:

$$\gamma = \frac{4q}{\pi r^3}, \qquad (8)$$

Based on the above, the predicted local shear rate was plotted while the blood vessel radius was swept from 0.1 to 2.5 mm, and the viscosity of the gel was swept from 0.025 to 10^5 Pa·s. The range of vessels sizes are classified into large (> 4 mm diameter), medium (1.5 ~ 4 mm diameter) and small (< 1.5 mm diameter) – for which corresponding arterial pressures of 80, 50, and 20 mmHg were assumed as they roughly correspond to major arteries, arterioles, and capillaries respectively. It has been reported that a shear rate of 5 s^{-1} can initiate thrombosis due to accumulation of factors, and thus this was used as the threshold for successful embolization.

S6. Additional information on PEGDA-nSi biocompatibility

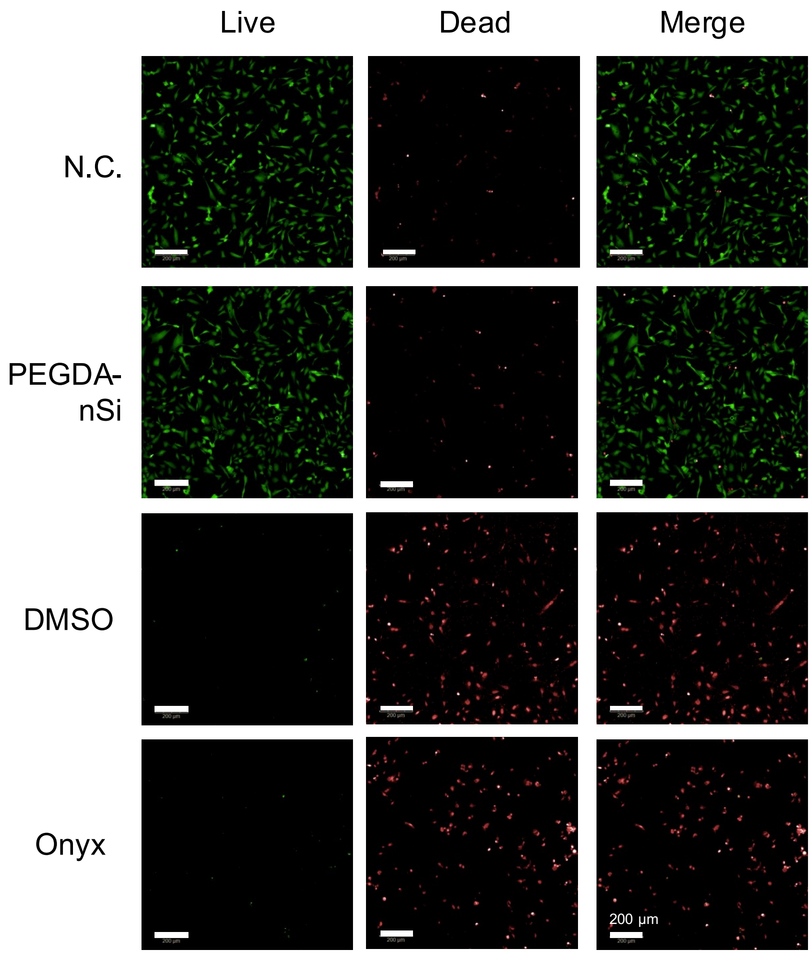

Figure S6. Fluorescent microscopic images of Live/Dead Assay of cells after indirect contact for 72 hours with Negative Control (blank), PEG-nSi, Onyx, or DMSO, again showing the high cytocompatibility of PEG-nSi compared to other agents. P.C. = positive control, N.C. = negative control. Scalebar = 200 um. *$p < 0.05$.

S7. Additional information on porcine preclinical model

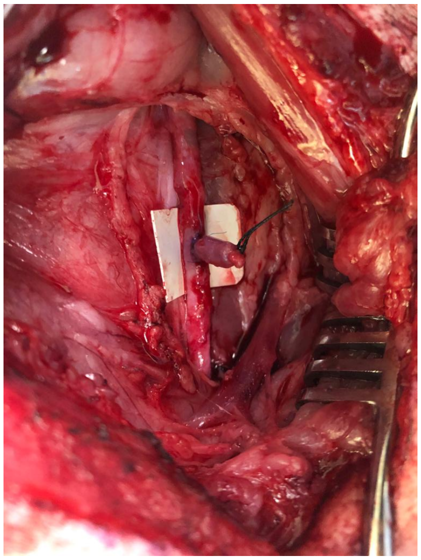

Figure S7. A photograph of carotid aneurysm created by venous pouch grafting method. A wide necked aneurysm was created on the left common carotid artery using the venous pouch microanastomosis method. A 1-cm portion of the left external jugular vein was harvested. The left common carotid artery was isolated at the mid-cervical region, temporary clips were applied, and a 5 mm arteriotomy was performed on the anterolateral wall. The venous pouch was then connected end-to-side to the arteriotomy.

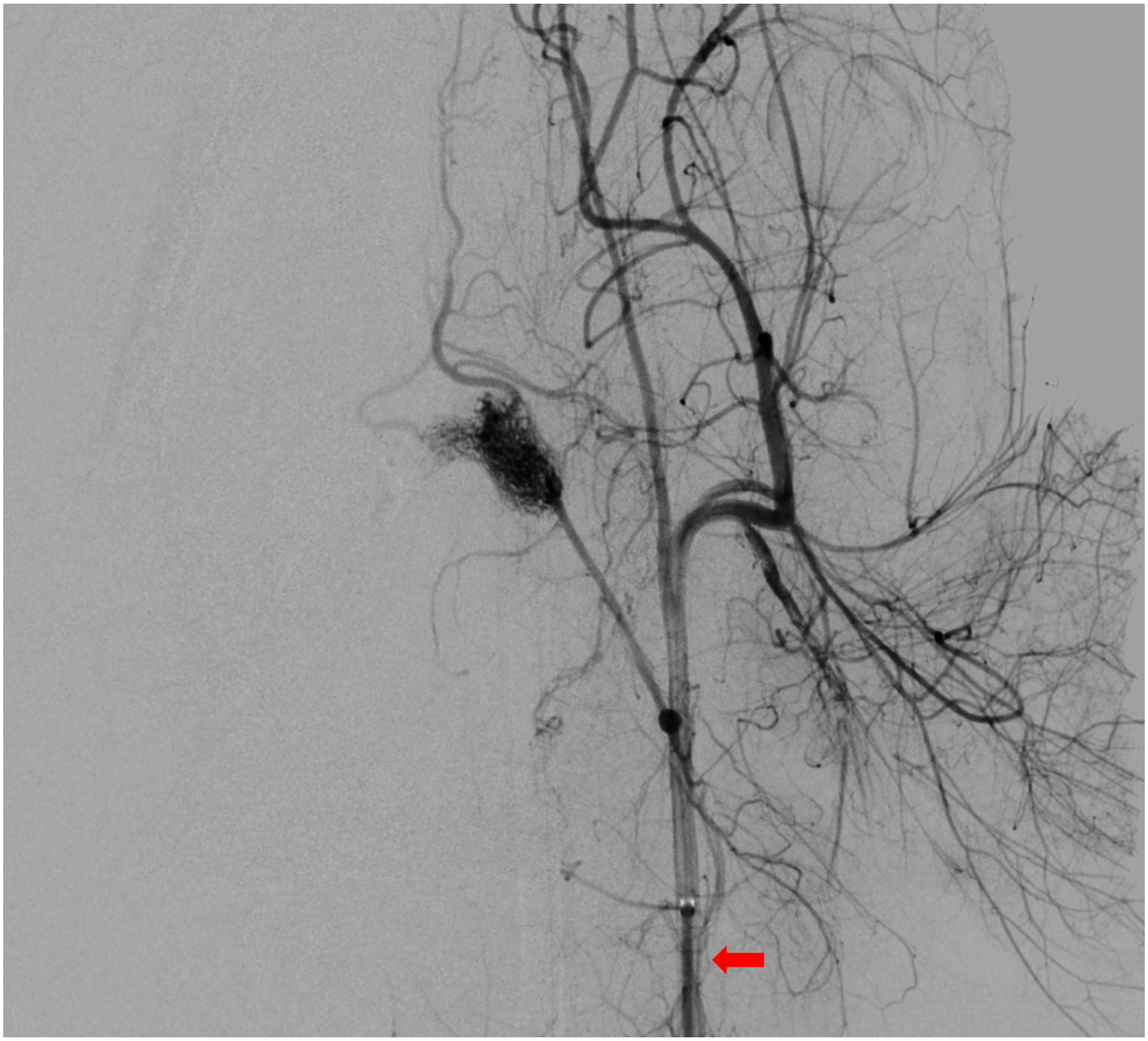

Figure S8. Post embolization angiogram of the common carotid artery, with the injection catheter just distal to the embolized aneurysm (red arrow), showing patency of the external carotid artery branches and the rete mirabile and downstream intracranial vessels.

S8. Description of supplementary videos

Video 1: A video demonstrating near instant on-demand switching between low viscosity injection and fully crosslinked extrusion using PEGDA-nSi hydrogel precursor in the custom catheter, based on XT27 2.7 French catheter (~0.8 mm diameter). Here, the delivered optical power at the tip of the optical fiber is 20 mW at 405 nm wavelength, while the flow rate is approximately 0.2 ml/min.

Video 2: Simulation of the viscosity and pressure change across the reaction chamber as a function of time. Surface plot represents the viscosity (scale bar in the units of Pa·s), while the contours represent the pressure (scale bar in the units of Pa). Note that due to the constant flow, the distal region within the reaction chamber begins to crosslink, but eventually reaches a steady state where most of the reaction chamber is filled with uniform viscosity precursor. Steady state is reached rapidly (300 ms), allowing precise operational control during embolization.

Video 3: A video of embolization procedure in a porcine inferior renal structure using PEGDA-nSi hydrogel and the custom catheter. The sequence of the video comprises of i) pre-treatment angiography, showing the overall vascular structure ii) hydrogel visualization during treatment iii) post treatment angiography, showing obliteration of the selected structure.

Video 4: A video of embolization procedure in a porcine rete mirabile structure using PEGDA-nSi hydrogel and the custom catheter. The sequence of the video comprises of i) pre-treatment angiography, showing the overall vascular structure ii) hydrogel visualization during treatment iii) post treatment angiography, showing obliteration of the selected structure.

Video 5: A video of embolization procedure in a porcine carotid aneurysm using PEGDA-nSi hydrogel and the custom catheter. The sequence of the video comprises of i) pre-treatment angiography, showing the overall vascular structure ii) hydrogel visualization during treatment iii) post treatment angiography, showing obliteration of the selected structure.

References:

[1] F. Arikan, D. Gandara, M. Esteves, A. Tomasello, J. Sahuquillo, *Interventional Neuroradiology* **2019**, *25*, 338.

[2] J. H. Lee, R. K. Prud'homme, I. A. Aksay, *Journal of Materials Research* **2001**, *16*, 3536.

[3] B. D. Fairbanks, M. P. Schwartz, C. N. Bowman, K. S. Anseth, *Biomaterials* **2009**, *30*, 6702.

[4] R. Brighenti, M. P. Cosma, L. Marsavina, A. Spagnoli, M. Terzano, *International Journal of Advanced Manufacturing Technology* **2021**, *117*, 481.
